# Antiproliferative activity of marine stingray *Dasyatis sephen* venom on human cervical carcinoma cell line

**DOI:** 10.1186/s40409-015-0036-5

**Published:** 2015-10-12

**Authors:** RK Rajeshkumar, R Vennila, S Karthikeyan, N Rajendra Prasad, M Arumugam, T Velpandian, T Balasubramaniam

**Affiliations:** 1 Ocular Pharmacology and Pharmacy, Centre for Ophthalmic Sciences, All India Institute of Medical Sciences (AIIMS), New Delhi, India; 2 Center of Advanced Study in Marine Biology, Annamalai University, Parangipettai Tamil Nadu, India; 3 Department of Biochemistry and Biotechnology, Annamalai University, Tamil Nadu, India

**Keywords:** Marine organisms, Membrane potential, Oxidative stress, ROS, Stingray, Venom

## Abstract

**Background:**

Venoms comprise mixtures of numerous bioactive compounds that have a wide range of pharmacologic actions. Toxins from venomous animals have attracted the attention of researchers because of their affinity for primary sites responsible for lethality and their efficacy at extremely low concentrations. The venoms of marine stingrays have not been extensively studied and limited data is available on them. The present study aims to evaluate the antiproliferative and biochemical properties of the venom obtained from a species of marine stingray (*Dasyatis sephen*) on human cervical cancer cell line HeLa.

**Methods:**

The antiproliferative effect of *D. sephen* venom was determined by MTT assay, and the oxidative stress was determined by lipid peroxidation method along with assessment of changes in the enzymatic and non-enzymatic antioxidant status. We observed intracellular reactive oxygen species (ROS) levels by DCFH-DA method, mitochondrial membrane potential alterations by rhodamine 123 staining and apoptotic morphological changes by acridine orange/ethidium bromide dual staining method.

**Results:**

*D. sephen* venom enhances lipid peroxidative markers such as thiobarbituric acid reactive substance, conjugated diene, and lipid hydroperoxide in HeLa cell lines. Stingray venom enhances the ROS levels, which is evidenced by the increased 2–7-diacetyl dichlorofluorescein fluorescence. Further, *D. sephen* venom treatment altered the mitochondrial membrane potential in HeLa cells. Additionally, we observed increased apoptotic morphological changes in *D. sephen* venom-treated groups.

**Conclusions:**

*Dasyatis sephen* venom exhibits potent antiproliferative effect on HeLa cell line and upon further purification it could be a promising antiproliferative agent.

**Electronic supplementary material:**

The online version of this article (doi:10.1186/s40409-015-0036-5) contains supplementary material, which is available to authorized users.

## Background

Marine organisms comprise approximately one-half of the total global biodiversity; therefore, they offer an important source for novel compounds that has been classified as the largest reservoir of natural molecules to be evaluated for drug activity [1]. A different type of environment exists in the ocean, where organisms live in competitive and aggressive surroundings that differ in many aspects from the terrestrial environment. This competitive environment demands the production of quite specific and potent active molecules by marine organisms [2]. These organisms have been continuously screened for pharmacologically active substances and till date over 6500 marine natural products have been isolated [3].

Cancer is characterized by the uncontrolled growth and spreading of abnormal cells. In normal cell function and tissue homeostasis, proliferation and apoptosis are balanced. Cancer cells display abnormal *in vivo* proliferation that is not balanced by compensatory apoptosis [4]. Apoptosis, defined as a controlled form of cell death, might represent a pivotal point in cancer treatment development [5]. For the past few decades, venom components have become the focus of researchers and have been extensively studied for their various anticancer properties with effective inhibition of proliferation. The anticancer potential of venom on adenocarcinoma cells was first reported for Naja snake venoms by Calmette et al. [6]. In addition, bee venom has also proven to have antiproliferative activity *in vitro* and ability to reduce tumor growth *in vivo* [7]. Scorpion venom toxins demonstrate antiproliferative activity against human glioma and leukemic cells as well [8, 9]. Marine environment is highly diverse and several compounds from marine origin have been approved for clinical use, including vidarabine (recurrent epithelial keratitis and superficial keratitis), cytarabine (cancer), ziconotide (chronic pain in cancer or AIDS), trabectedin (soft tissue sarcoma) and halaven (metastatic breast cancer) [10, 11]. But, concerning fish venoms, limited data are available about their bioactive potential.

Marine stingrays are venomous fish of the Elasmobranch family and are mainly found in temperate and tropical areas of the world. Stingrays are considered to be one of the most significant venomous fish in the world [12]. They have serrated spines at the base of the tail that penetrate the body of the victim. The epidermal covering of the spine releases venom into the sting site causing severe pain and tissue necrosis [13]. Stingray venom exhibits neurotoxic, cardiotoxic, fibrinogenolytic and anticoagulant activities and are composed of proteins, serotoxin, vasoconstrictor peptides and several other unidentified components [14–18]. In this study, the mechanism by which the venom of the marine stingray *D. sephen* inhibits cell proliferation and the associated apoptotic pathways are examined in human cervical cancer cells (HeLa).

## Methods

### Chemicals

Bovine serum albumin, thiobarbituric acid (TBA), phenazine methosulphate (PMS), nitroblue tetrazolium (NBT), 5,5-dithiobis(2-nitrobenzoic acid) (DTNB), 3-(4,5-dimethyl-2-thiaozolyl)-2,5-diphenyl-2H-tetrazolium bromide (MTT), 2–7-diacetyl dichlorofluorescein (DCFH-DH), rhodamine 123 (Rh-123), acridine orange, ethidium bromide, heat inactivated fetal calf serum (FCS), minimum essential medium (MEM), McCoy’s modified medium, glutamine, penicillin-streptomycin, EDTA and trypsin were purchased from Sigma Chemicals Co. (USA).

### Venom extraction

Stingray specimens were collected from Parangipettai coast (11°30’1.19“N 79°46’20.50”E, Tamil Nadu, India) by local fishermen. The spines that are usually discarded by fishermen due to the lack of commercial value were removed from the base of the tail and transported to the laboratory in an icebox. The tissue covering the stingers was scratched and homogenized in PBS buffer, pH 7.4 and centrifuged at 5000 × *g* for 10 min. The supernatant (venom) was stored at − 20 °C until use [19]. Protein concentration of the supernatant was estimated by standard method using BSA as protein standard. Details about the *D. sephen* and stinger images are provided in Additional file 1.

### Cell lines and culture conditions

The present work was carried out on human cervical cancer cell line (HeLa). The cell line was obtained from the National Centre for Cell Science (NCCS), Pune, India. HeLa cells were grown as monolayer in MEM medium supplemented with 10 % FCS, 2mM glutamine, and 100 U/mL penicillin-streptomycin at 37 °C in 5 % CO_2_ atmosphere. Stocks were maintained in 25 cm^2^ tissue culture flasks.

### Study group and dose fixation study

Cells were treated with different concentrations of stingray venom (2, 4, 8, 12, 16, 20 or 24 μg/mL) and the cytotoxicity was observed by MTT assay. PBS used as a sham control. The results assessed by MTT assay were employed in further experiments in which five groups were defined based on the venom administration doses:

- Group I: untreated HeLa cells (control)
- Group II: HeLa cells + *D. sephen* venom (4 μg/mL)
- Group III: HeLa cells + *D. sephen* venom (8 μg/mL)
- Group IV: HeLa cells + *D. sephen* venom (12 μg/mL)
- Group V: HeLa cells + *D. sephen* venom (16 μg/mL)

### Drug sensitivity assay

The growth inhibitory activity of cells was determined by the MTT assay based on the detection of mitochondrial dehydrogenase activity in living cells [20]. Each culture well was incubated for 24 h with different doses of *D. sephen* venom. Ten microliters of MTT solution (5 mg/mL in PBS) was added and incubated for 4 h to allow color development. An equal volume of DMSO was then added to stop the reaction and to solubilize the blue crystals formed. The absorbance was taken at the wavelength of 570 nm.

### Measurement of intracellular ROS in cells by spectrofluorimetric and fluorescence microscopic methods

Intracellular ROS was measured by using a nonfluorescent probe, DCFH-DA, which penetrates into the intracellular matrix of cells to be oxidized by ROS to fluorescent dichlorofluorescein (DCF) [21]. Cells were incubated for 24 h with different concentrations of *D. sephen* venom. Fluorescent dye DCFH-DA was then added to the cells and incubated for 30 min. The cells were washed with PBS to remove the excess dye before fluorescent measurements that were carried out with excitation and emission filters set at 485 ± 10 and 530 ± 12.5 nm, respectively (Shimadzu RF-5301 PC Spectrofluorometer, Japan). Fluorescence microscopic images were taken using blue filter (450–490 nm) (Nikon, Eclipse TS100, Japan).

### Alterations in mitochondrial membrane potential

After cell incubation with *D. sephen* venom for 24 h, fluorescent dye Rh-123 (10 μg/mL) was added to the cells. The cells were then incubated for 30 min, washed with PBS and analyzed in fluorescence microscope using blue filter [21]. Polarized mitochondria were marked by orange-red fluorescence, and depolarized mitochondria were marked by green fluorescence.

### Apoptotic morphological changes by acridine orange-ethidium bromide dual staining method

Staining of DNA with acridine orange (AO) and ethidium bromide (EBr) allowed visualization of the condensed chromatin of dead apoptotic cells [21]. Stained cells were viewed under a fluorescence microscope. The number of cells showing features of apoptosis was counted as a function of the total number of cells present in the field.

### Estimation of lipid peroxidation markers

The cells were harvested by trypsinization. The pellet obtained was suspended in PBS and taken for the measurement of lipid peroxidative markers such as thiobarbituric acid reactive substances (TBARS), conjugated dines (CD), and lipid hydro peroxide (LHP), according to the procedures described elsewhere [22–24].

### Estimation of antioxidant enzyme activity

The supernatant obtained after centrifuging the trypsinized cells was used for the measurement of activities of antioxidant enzymes, superoxide dismutase (SOD), catalase (CAT), and glutathione peroxidase (GPx), according to the procedures described elsewhere [25–27]

### Estimation of reduced glutathione levels

The levels of reduced glutathione (GSH) were determined in the supernatant obtained after centrifuging the trypsinized cells according to the procedures described elsewhere [28].

### Statistical analysis

Statistical analysis was performed using one way analysis of variance (ANOVA) followed by Duncan’s multiple range test (DMRT) by using Statistical Package of Social Science (SPSS) version 12.0 for Windows. The values were expressed as mean ± SD for six samples in each group. In addition, *p* value of < 0.05 was considered as statistically significant.

## Results

### Effect of *D. sephen* venom on cell proliferation

**Fig. 1:**
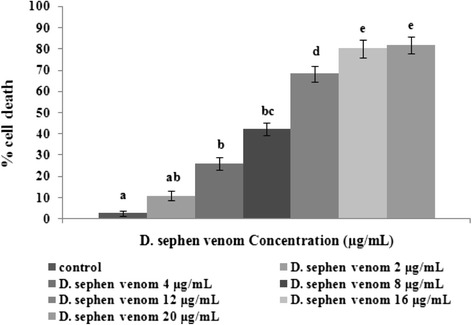
Cytotoxicity of *D. sephen* venom on HeLa cells. The venom was incubated with cancer cells for 24 h and cytotoxicity was observed by MTT assay. Cell death was observed in a concentration-dependent manner (2–20 μg/mL) in HeLa cells. Values were given as mean ± SD of six experiments in each group. Values not sharing the same letter differ significantly (*p* < 0.05) from control (DMRT)

Effect of *D. sephen* venom on cell proliferation was determined by MTT assay. The proliferation of HeLa cells was significantly inhibited by *D. sephen*venom. Figure 1 shows the changes in the percentage of cell death in control and venom-treated cells. The treatment with 2 μg/mL of *D. sephen* venom did not show significant (*p* < 0.05) proliferation inhibition. The treatment with 4, 8, 12 and 16 μg/mL of *D. sephen* venom significantly inhibited in HeLa cells. Concentrations of 16 and 20 μg/mL displayed almost the same inhibitory effect (80 and 81 % inhibition) on HeLa cells. Hence, for further tests *D. sephen* venom concentrations of 4, 8, 12 and 16 μg/mL were used on HeLa cells.

### *D. sephen* venom generates intracellular ROS

**Fig. 2:**
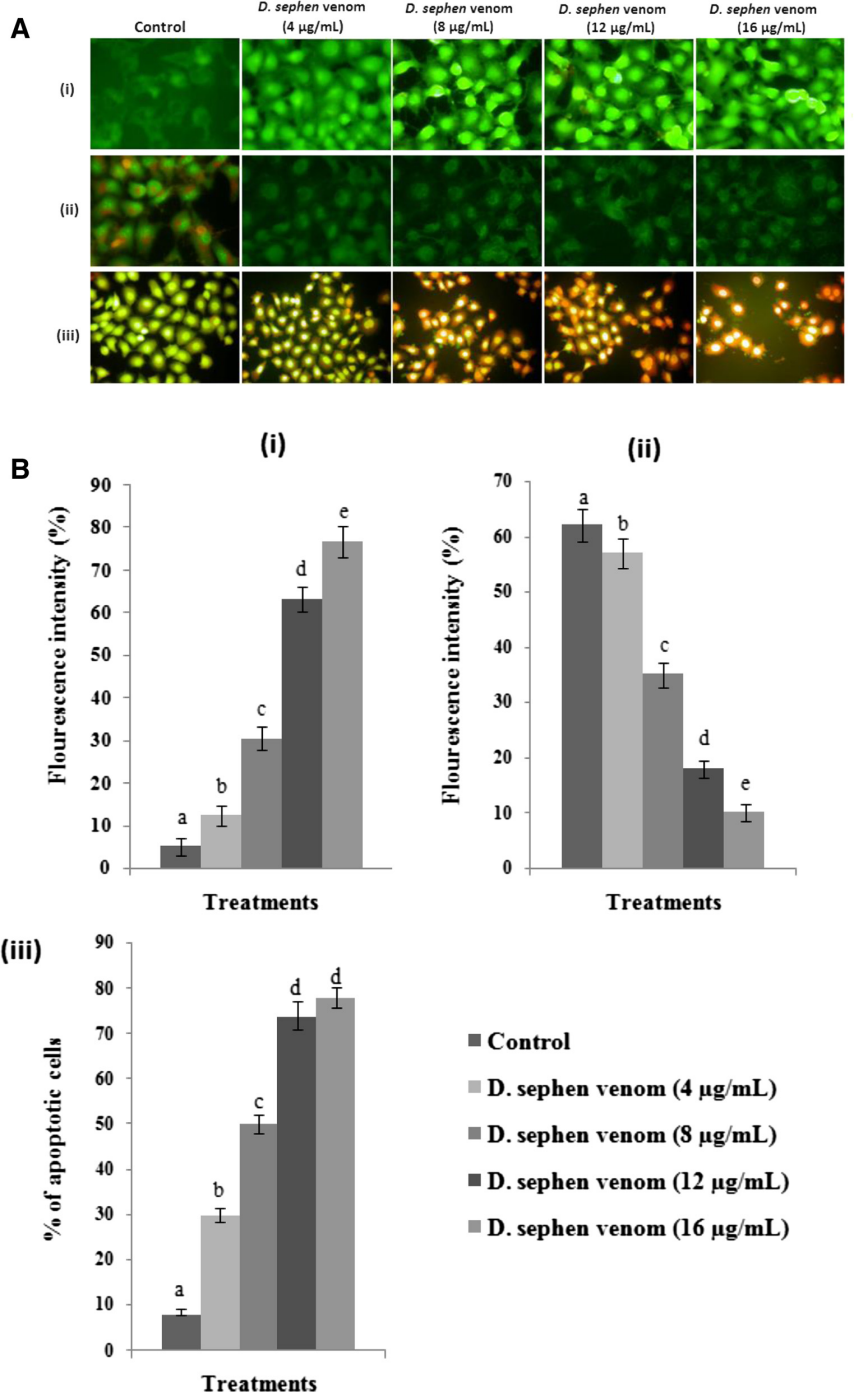
**a** Photomicrographs show the effect of *D. sephen* venom on: (i) intracellular ROS generation; (ii) mitochondrial membrane potential and; (iii) apoptotic morphology changes in HeLa cells. **b** Effect of *D. sephen* venom on: (i) intracellular ROS generation; (ii) mitochondrial membrane potential and; (iii) percentage of apoptosis in HeLa cells. Values were given as mean ± SD of six experiments in each group. Bars not sharing a common letter differ significantly (*p* < 0.05) from control (DMRT)

Levels of ROS in control and *D. sephen* venom-treated cells are depicted in Fig. 2 – A (i) and B (i). Venom treatment significantly increased ROS level in HeLa cells. Venom concentrations of 4, 8, 12, and 16 μg/mL increased ROS levels to 12, 30, 63 and 75 %, respectively, when compared to control group. Among all tested doses, 16 μg/mL showed maximum generation of ROS in HeLa cells.

### *D. sephen* venom modulates mitochondrial membrane potential

Changes in mitochondrial membrane potential in control and *D. sephen* venom-treated cells are depicted in Fig. 2 – A (ii) and B (ii). *D. sephen* venom treatment significantly increased mitochondrial depolarization in HeLa cells. After 24-hour incubation, venom treated cells showed significantly decreased mitochondrial membrane potential to 57, 35, 18, and 10 % at 4, 8, 12, and 16 μg/mL concentrations of *D. sephen* venom in HeLa cells, respectively, when compared to control group. Among all the doses tested, 16 μg/mL of *D. sephen* venom showed the highest level of mitochondrial depolarization in HeLa cells. Polarized mitochondria were marked by orange-red fluorescence and depolarized mitochondria were marked by green fluorescence.

### Effect of *D. sephen* venom on apoptotic morphological changes

Figure 2 – A (iii) and B (iii) show the effect of *D. sephen* venom on apoptotic morphological changes. Figure 2 – A (iii) shows the fluorescence microscopic observation of the untreated and treated cancer cells. Untreated cancer cells appeared in green (AO stained) whereas venom treated cells appeared in red/orange (EtBr stained) that revealed the presence of apoptotic cells. Figure 2 – B (iii) shows the percentage of apoptosis. Venom concentrations of 4, 8, 12, and 16 μg/mL increased apoptotic cells levels to 30, 50, 74 and 78 %, respectively, when compared to the control group. Among all the doses tested, 16 μg/mL of *D. sephen* venom showed increased apoptotic levels in HeLa cells.

### Changes in the levels of lipid peroxidative markers and the activities of enzymatic antioxidants

**Fig. 3:**
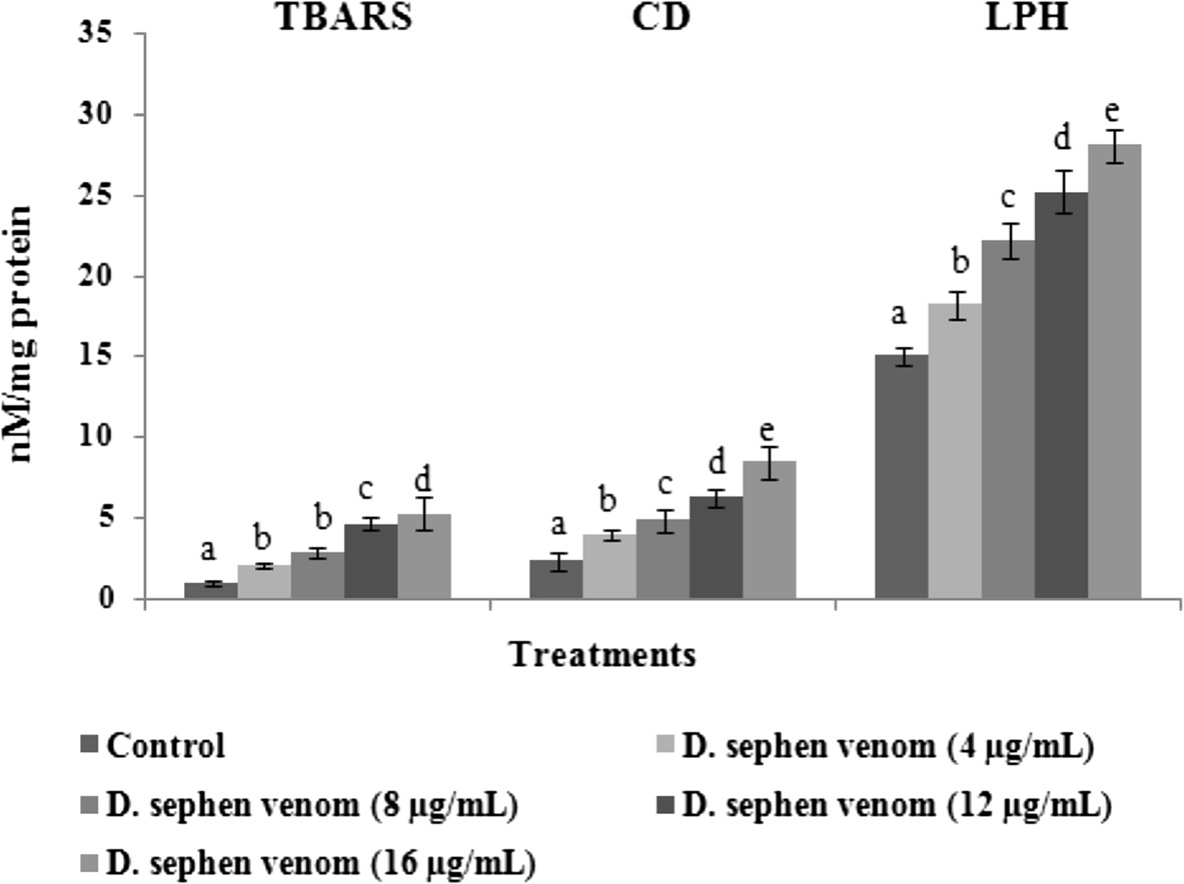
Effect of *D. sephen* venom on lipid peroxidation in HeLa cells. Bars represent the changes on the levels of lipid peroxidative markers (TBARS, CD and LPH) in HeLa cells. Values were given as mean ± SD of six experiments in each group. Values not sharing the same letter differ significantly (*p* < 0.05) from control (DMRT)

We observed the levels of lipid peroxidative markers, such as TBARS, CD and LHP in control and venom-treated cells (Fig. 3). *D. sephen* venom treatment increased the levels of lipid peroxidation in Hela cells. Among all the concentrations (4, 8, 12, and 16 μg/mL) tested, 16 μg/mL significantly increased levels of TBARS, CD and LHP in HeLa cells.

**Fig. 4:**
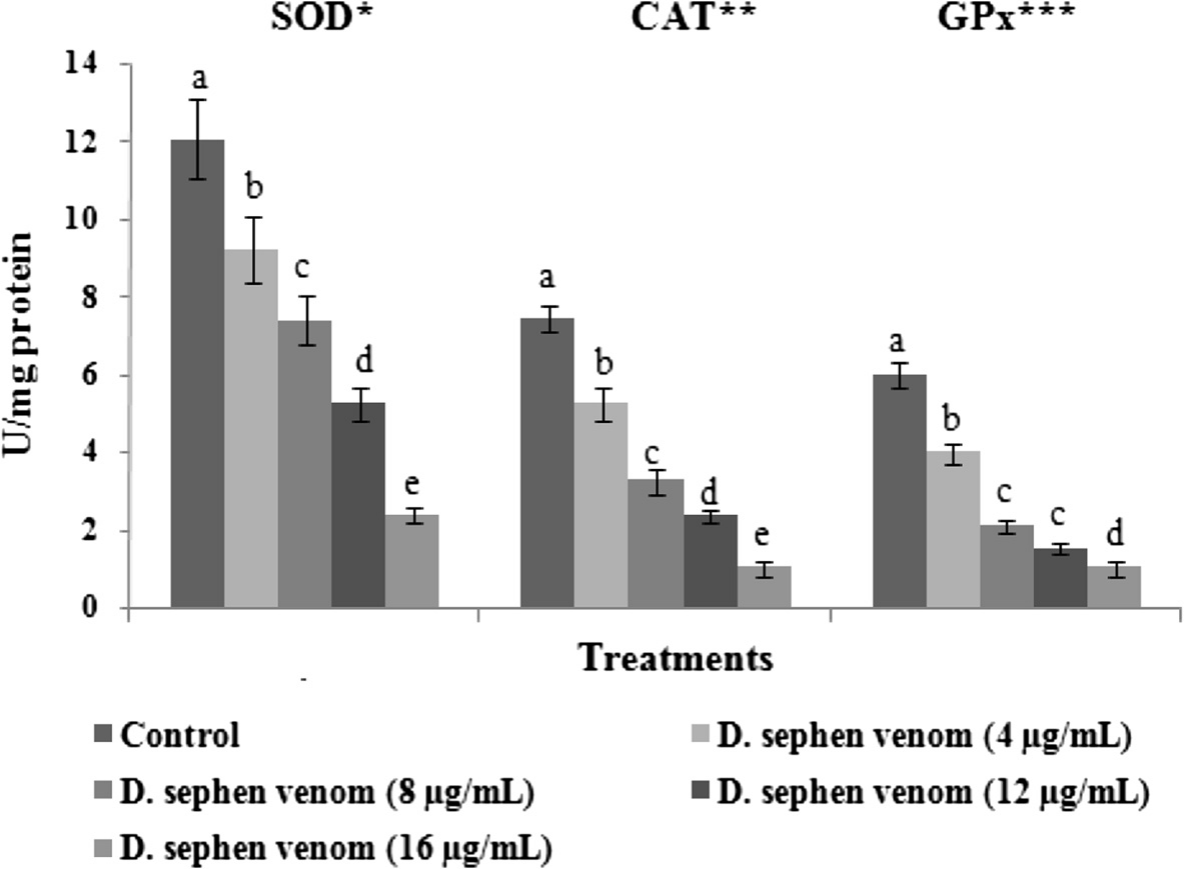
Effect of *D. sephen* venom on cellular antioxidant status in HeLa cells. Bars represent the changes on the activities of SOD, CAT and GPx in control and venom treated cells. *Enzyme concentration required for 50 % inhibition of nitroblue tetrazolium reduction in one minute. **μmol of hydrogen peroxide consumed per minute. ***μg of glutathione consumed per minute

**Fig. 5:**
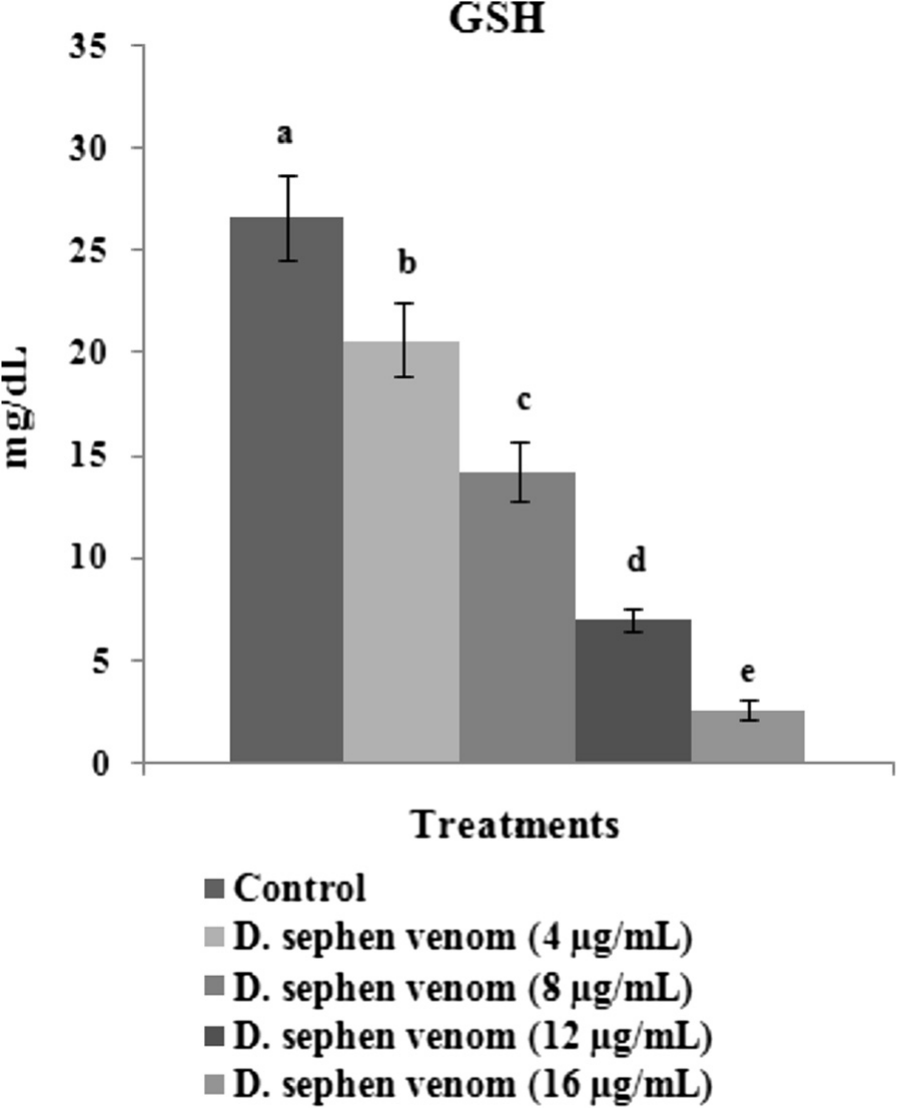
Effect of *D. sephen* venom on reduced GSH levels in HeLa cells. Treated cells were incubated for 24 h at 37 °C and then reduced GSH levels were measured in cell lysate. Values were given as mean ± SD of six experiments in each group. Values not sharing the same letter differ significantly (*p* < 0.05) from control (DMRT)

Activities of enzymatic antioxidants such as SOD, CAT, and GPxare are depicted in Fig. 4. *D. sephen* venom (4, 8, 12, and 16 μg/mL) treatment significantly decreased the activities of SOD, CAT, and GPx in HeLa cells. Among all tested doses, 16 μg/mL of *D. sephen* venom significantly decreased enzymatic activities when compared with other doses in HeLa cells. In this study, the effect of *D. sephen* venom on glutathione levels in venom-treated cancer cells was examined. Levels of GSH in control and venom-treated cells are showed in Fig. 5. The treatment of *D. sephen* venom (4, 8, 12, and 16 μg/mL) decreased GSH levels in HeLa cells. Regarding all the doses tested, 16 μg/mL of *D. sephen* venom significantly decreased GSH levels in HeLa cells.

## Discussion

Venoms from many marine sources have demonstrated to be highly effective in low concentrations. Marine fish venoms have not been fully explored and their biological potentials have not been completely characterized. In the present study, we evaluated in vitro antiproliferative potential of stingray venom extracts on HeLa cell line. *D. sephen* venom venom significantly decreased the viability of cancer cells. There was a significant increase in cytotoxicity (26, 42, 68 and 80 %) of HeLa cells with increasing concentrations of this venom at 4, 8, 12, and 16 μg/mL, respectively. Our results indicate that concentrations of the compounds play a vital role in cytotoxicity. It is very likely that *D. sephen* venom may disrupt mitochondrial dehydrogenase activity of cancer cells at higher concentrations [29]. Mitogenic and cytotoxic effects of venom from marine sources such as *S. verrucosa* and *H. rubripinnis* on regular and tumor cell lines have been reported earlier [30]. However, the molecular role of venom in disrupting intracellular signaling pathways leading to apoptosis (programed cell death) of cancer cells have not yet been elucidated [31]. Fleury et al. [32] stated that an increase in the production of ROS associated with disturbance in the oxidative status results in the impairment of mitochondrial function that triggers the apoptosis pathway. Hence, in this study, assessment of ROS species, mitochondrial function and antioxidant enzyme activity determination was carried out.

ROS is known to be generated intracellularly though three pathways: as the byproduct of normal aerobic metabolism; as second messengers in various signal transduction pathways; and in response to environmental stress. Depending upon the concentration, ROS is known to elicit a wide spectrum of biological responses ranging from mitogenic to proliferative effects at low concentrations, and macromolecular damage leading to cell death at high concentrations [33]. Previous reports have suggested that Okinawa habu apoxin protein-1 (OHAP-1) from Okinawa habu (*T. flavovirudis*) venom induced apoptosis in malignant glioma cell lines by promoting the generation of intracellular ROS [34]. Similarly, in our study *D. sephen* venom caused a rapid increase of intracellular ROS levels (63 and 76 % at 12 and 16 μg/mL concentrations, respectively) in HeLa cells after 24 h of incubation.

ROS are known to be generated from the reaction of leaked electrons with oxygen under various systems including lipid peroxidation. Lipid peroxidation is a multistep process in which the initially formed lipid radicals are converted to TBRS via the unstable intermediate products CD and LPH [35]. We observed the levels of lipid peroxidative products, such as TBARS, CD and LHP in control and *D. sephen* venom-treated cells. *D. sephen* venom treatment increased the levels of lipid peroxidative products in HeLa cells. Among all the concentrations tested (4, 8, 12, and 16 μg/mL), 16 μg/mL of venom showed significantly increased levels of TBARS, CD and LHP in HeLa cells.

Mitochondrion is one of the most important organelles that regulate cell death and mark apoptosis [36]. Functional alterations of mitochondria have been shown to play an important role in cell apoptosis [37]. The mitochondria of normal cells pump H^+^ from initial ground substance to the outside of the endomembrane creating a transmembrane potential. In the present study, mitochondria membrane potential was calculated in order to examine whether apoptosis is accompanied by the loss of mitochondrial transmembrane potential. We observed *D. sephen* venom-treated cells showed significantly decreased mitochondrial membrane potential by 57, 35, 18, and 10 % at concentrations of 4, 8, 12, and 16 μg/mL, respectively, in HeLa cells when compared to the control group. Rh-123, a mitochondria-specific membrane permeable dye, was accumulated in the mitochondria of control cells whereas venom-treated cells exhibited decreased uptake of it with increasing concentrations. Our results indicate that the mithochondrial damage occurred during *D. sephen* venom treatment, which suggests that the alteration of mitochondrial membrane potential (MMP) may have a role in venom-induced cell death. HeLa cell death induced by venom might be due to mitochondrial toxicity, as the MMP collapsed before apoptosis. This observation corroborates a previous study in which treatment with the *O. doriae* venom induced reactive nitrogen intermediates, caspase 3 and depolarization in mitochondria [38].

Apoptosis plays an important role in determining cellular cytotoxicity following drug treatment [39]. It is known to be associated with a characteristic set of morphological features, including membrane blabbing, chromosomal condensation, nuclear fragmentation, cell shrinkage and formation of apoptotic bodies. In the present study, we observed that *D. sephen* venom pretreatment significantly increased morphological changes associated in HeLa cells leading to 74 to 78 % of apoptosis at concentrations of 12 to 16 μg/mL. Fluorescence microscopic observation of the cells showed a typical apoptotic morphology – cell pyknosis, chromosome condensation and nuclear fragmentation in *D. sephen* venom-treated cells when stained with EtBr/AO.

Acridine orange (AO) is a cationic dye known to penetrate the intact membrane of living cells and stain DNA. On the other hand, EtBr is not incorporated by live cells. Hence, control cells can be observed as green under blue emission. On the contrary, cells undergoing apoptosis are not able to exclude EtBr and then exhibit more intense red color [40]. Increased ROS levels and loss of mitochondrial membrane potential might be the reason for increased apoptotic morphological changes such as cell pyknosis and chromosome condensation observed in the venom-treated cells.

In our body, antioxidant enzymatic defense is a very important tool to neutralize oxygen free radical-mediated tissue injury [41]. Free radical scavenging enzymes such as CAT, SOD, and GPx are the first line of cellular defense against oxidative injury, decomposing O_2_ and H_2_O_2_ before their interaction to form the more reactive hydroxyl radical. Antioxidant enzymes can antagonize initiation and promotion phases of carcinogenesis and their levels are reduced in many malignancies [42]. Cell malignancy or transformation is often accompanied by decrease in activity of antioxidant enzymes like CAT, SOD, and GPx, which increases the cell sensitivity to prooxidant compounds [43]. *D. sephen* venom at concentrations of 4, 8, 12, and 16 μg/mL significantly decreased the activities of SOD, CAT, and GPx in HeLa cells. Among all the doses tested, 16 μg/mL expressively decreased enzymatic activities when compared with other doses in HeLa cells. It has been shown in the literature that anticancer agents deplete intracellular antioxidants and because of that, there is increased accumulation of free radicals inside the cells which modulate the opening of the mitochondrial permeability transition pore resulting in apoptosis [21, 35]. The present finding indicates that the *D. sephen* venom has caused cell death by damaging mitochondria, increasing intracellular ROS formation and depletion of cellular antioxidant enzyme in a dose-dependent manner in HeLa cells. This corroborates a previous study that showed the cytotoxic potential of *Conus vexillum* venom, which induced oxidative stress [44].

GSH plays a key role in protecting cells from electrophilic compounds and free radicals generated during cellular metabolism. Depletion of GSH can lead to tumor cell death in vitro, especially in melanocytic cells that generate high levels of oxyradicals [45, 46]. In this study, the effect of *D. sephen* venom on reduced glutathione levels in HeLa cancer cells was examined. The treatment with venom (4, 8, 12, and 16 μg/mL) decreased GSH levels in HeLa cells. Among all the doses tested, 16 μg/mL significantly decreased GSH levels in HeLa cells. The resistance in most cases is associated with higher GSH levels within these cancer cells. Thus, approaches to cancer treatment could potentially benefit from a selective GSH depleting strategy [47]. Therefore, our results indicate that the prominent decrease of GSH levels in cancer cells treated with *D. sephen* venom, and further purification of its active constituents could lead to a novel drug candidate.

## Conclusions

The present findings suggest that *D. sephen* venom initiates cancer cell death by decreasing cell proliferation, antioxidant status and mitochondrial membrane potential; and by increasing intracellular ROS, lipid peroxidation and apoptosis in human cervical (HeLa) cancer cells. The current results clearly demonstrate the involvement of an oxidative mechanism for the antiproliferative effect of *D. sephen* venom on HeLa cells. Thus, marine stingray venoms could be used as a source of antiproliferative agents after further purification in the future.

## Additional file

Additional file 1: **Scientific classification and image of*D. sephen.*** (DOCX 105 kb)

## References

[1] de Vries DJ, Beart PM (1995). Fishing for drugs from the sea: status and strategies. Trends Pharmacol Sci.

[2] Aneiros A, Garateix A (2004). Bioactive peptides from marine sources: pharmacological properties and isolation procedures. J. Chromatogr. B Anal. Technol. Biomed. Life Sci.

[3] Bhakuni DS (1998). Some aspects of bioactive marine natural products. J Indian Chem Soc.

[4] Weinstein IB (2000). Disorders in cell circuitry during multistage carcinogenesis: the role of homeostasis. Carcinogenesis.

[5] Kim MS, Kang HJ, Moon A (2001). Inhibition of invasion and induction of apoptosis by curcumin in H-ras-transformed MCF10A human breast epithelial cells. Arch Pharm Res.

[6] Calmette A, Saenz A, Costil L (1933). Effets du venin de cobra sur les greffes cancereuses et sur le cancer spontane (adenocarcinoma) de la souris. CR Acad Sci.

[7] Orsolic N, Sver L, Vestovsek S, Terzic S (2003). Inhibition of mammary carcinoma cell proliferation in vitro and tumor growth in vivo by bee venom. Toxicon.

[8] Wang WX, Ji YH (2005). Scorpion venom induces glioma cell apoptosis in vivo and inhibits glioma tumor growth in vitro. J. Neurooncol.

[9] Gupta SD, Gomes A, Debnath A, Saha A, Gomes A (2003). Apoptosis induction in human leukemic cells by a novel protein Bengalin, isolated from Indian black scorpion venom: through mitochondrial pathway and inhibition of heat shock proteins. Chem Biol Interact.

[10] Mayer AM, Glaser KB, Cuevas C, Jacobs RS, Kem W, Little RD (2010). The odyssey of marine pharmaceuticals: a current pipeline perspective. Trends Pharmacol Sci.

[11] Blunt JW, Copp BR, Keyzers RA, Munro MH, Prinsep MR (2013). Marine natural products. Nat Prod Rep.

[12] Junghanss T, Bodio M (2006). Medically important venomous animals: biology, prevention, first aid, and clinical management. Clin Infect Dis.

[13] Fenner PJ, Williamson JA, Skinner RA (1989). Fatal and non-fatal stingray envenomation. Med J Aust.

[14] Conceição K, Konno K, Melo RL, Marques EE, Hiruma-Lima CA, Lima C (2006). Orpotrin: a novel vasoconstrictor peptide from the venom of the Brazilian stingray Potamotrygon gr. orbignyi. Peptides.

[15] Conceição K, Santos JM, Bruni FM, Klitzke CF, Marques EE, Borges MH (2009). Characterization of a new bioactive peptide from Potamotrygon gr. Orbignyi freshwater stingray venom. Peptides.

[16] Lalwani K (1995). Animal toxins: scorpaenidae and stingrays. Br J Anaesth.

[17] Weiss BF, Wolfenden DH (2001). Survivor of a stingray injury to the heart. Med J Aust.

[18] Kumar KR, Vennila R, Kanchana S, Arumugam M, Balasubramaniam T (2011). Fibrinogenolytic and anticoagulant activities in the tissue covering the stingers of marine stingrays Dasyatis sephen and Aetobatis narinari. J Thromb Thrombolysis.

[19] Haddad Junior V, Neto GD, de Paula Neto JB, de Luna Marques FP, Barbaro CK (2004). Freshwater stingrays: study of epidemiologic, clinic and therapeutic aspects based on 84 envenomings in humans and some enzymatic activities of the venom. Toxicon.

[20] Mosmann T (1983). Rapid colorimetric assay for cellular growth and survival: application to proliferation and cytotoxicity assay. J. Immunol. Method.

[21] Karthikeyan S, Prasad NR, Ganamani A, Balamurugan E (2013). Anticancer activity of resveratrol-loaded gelatin nanoparticles on NCI-H460 non-small cell lung cancer cells. Biomed Prev Nutr.

[22] Niehaus WG, Samuelsson B (1968). Formation of malonaldehyde from phospholipid arachidonate during microsomal lipid peroxidation. Eur J Biochem.

[23] Klein RA (1970). The detection of oxidation in liposome preparations. Biochem Biophys Acta.

[24] Jiang ZY, Hunt JV, Wolff SP (1992). Ferrous ion oxidation in the presence of xylenol orange for detection of lipid hydroperoxide in low density lipoprotein. Anal Biochem.

[25] Kakkar ZYP, Das B, Viswanathan PN (1984). A modified spectrophotometric assay of superoxide dismutase (SOD). Indian J Biochem Biophys.

[26] Sinha KA (1972). Colorimetric assay of catalase. Anal Biochem.

[27] Rotruck JT, Pope AL, Ganther HE, Swanson AB, Hafeman DG, Hoekstra WG (1973). Selenium: biochemical role as a component of glutathione peroxidase. Science.

[28] Ellman GL (1959). Tissue sulfhydryl groups. Arch Biochem Biophys.

[29] Mignotte B, Vayssiere JL (1998). Mitochondria and apoptosis. Eur J Biochem.

[30] Satoh F, Nakagawa H, Yamada H, Nagasaka K, Nagasaka T, Araki Y (2002). Fishing for bioactive substances from scorpionfish and some sea urchins. J. Nat. Toxins.

[31] Balasubashini MS, Karthigayan S, Somasundaram ST, Balasubramanian T, Viswanathan P, Menon VP (2006). In vivo and in vitro characterization of the biochemical and pathological changes induced by lionfish [Pterois volitans] venom in mice. Toxicol Mech Methods.

[32] Fleury C, Mignotte B, Vayssiére JL (2002). Mitochondrial reactive oxygen species in cell death signaling. Biochimie.

[33] Martindale JL, Holbrook NJ (2002). Cellular response to oxidative stress: signaling for suicide and survival. J Cell Physiol.

[34] Sun LK, Yoshii Y, Hyodo A, Tsurushima H, Saito A, Harakuni T (2003). Apoptotic effect in the glioma cells induced by specific protein extracted from Okinawa Habu (Trimeresurus flavoviridis) venom in relation to oxidative stress. Toxicol in Vitro.

[35] Prasad NR, Karthikeyan A, Karthikeyan S, Reddy BV (2011). Inhibitory effect of caffeic acid on cancer cell proliferation by oxidative mechanism in human HT-1080 fibrosarcoma cell line. Mol Cell Biochem.

[36] Hildeman DA, Mitchell T, Teague TK, Henson P, Day BJ, Kappler J (1999). Reactive oxygen species regulate activation-induced T cell apoptosis. Immunity.

[37] Chou DA, Kuo YH, Jan MS, Chang YY, Chen YC, Chiu HL (2012). Caffeate derivatives induce apoptosis in COLO 205 human colorectal carcinoma cells through Fas- and mitochondria-mediated pathways. Food Chem.

[38] Zargan J, Sajad S, Umar M, Naime M, Ali S, Khan HA (2011). Scorpion (Odontobuthus doriae) venom induces apoptosis and inhibits DNA synthesis in human neuroblastoma cells. Mol Cell Biochem.

[39] Zhang L, Li H, Wu WT (2004). Purification and characterization of cytotoxins from Agkistrodon acutus venom and their anticancer activity. J. Chin. Pharm. Sci.

[40] Belloc F, Dumain P, Boisseau MR, Jalloustre C, Reiffers J, Bernard P (1994). A flow cytometric method using Hoecht 33342 and propidium Iodide for simultaneous analysis and apoptosis determination in unfixed cells. Cytometry.

[41] Polidoro G, Di Ilio C, Arduini A, La Rovere G, Federici G (1984). Superoxide dismutase, reduced glutathione and TBA-reactive products in erythrocytes of patients with multiple sclerosis. Int J Biochem.

[42] Matés JM, Sánchez-Jiménez FM (2000). Role of reactive oxygen species in apoptosis: implications for cancer therapy. Int J Biochem Cell Biol.

[43] Abdel-Rahman MA, Abdel-Nabi IM, El-Naggar MS, Abbas OA, Strong PN (2013). Conus vexillum venom induces oxidative stress in Ehrlich’s ascites carcinoma cells: an insight into the mechanism of induction. J. Venomous Anim. Toxins Incl. Trop. Dis.

[44] Sergediene E, Jonsson K, Szymusiak H, Tyrakowska B, Rietjens IM, Cenas N (1999). Prooxidant toxicity of polyphenolic antioxidants to HL-60 cells: description of quantitative structure activity relationships. FEBS Lett.

[45] Estrela JM, Ortega A, Obrador E (2006). Glutathione in cancer biology and therapy. Crit Rev Clin Lab Sci.

[46] Ortega AL, Carretero J, Obrador E, Gambini J, Asensi M, Rodilla V (2003). Tumor cytotoxicity by endothelial cells. Impairment of the mitochondrial system for glutathione uptake in mouse B16 melanoma cells that survive after in vitro interaction with the hepatic sinusoidal endothelium. J Biol Chem.

[47] Vad NM, Yount G, Moore D, Weidanz J, Moridani MY (2009). Biochemical mechanism of acetaminophen (APAP) induced toxicity in melanoma cell lines. J Pharm Sci.

