# Erratum: Antiproliferative activity of marine stingray *Dasyatis sephen* venom on human cervical carcinoma cell line

**DOI:** 10.1590/s40409-015-0036-5er

**Published:** 2020-07-10

In the article entitled “Antiproliferative activity of marine stingray *Dasyatis sephen* venom on human cervical carcinoma cell line”, DOI: 10.1186/s40409-015-0036-5, published in *Journal of Venomous Animals and Toxins including Tropical Diseases*, 2015, 21:41, page 5:

Figure 2A inadvertently contained a mistake. The second panel of line (ii) is the same of the third one, of column “*D. sephen* venom (8 µg/mL)”:


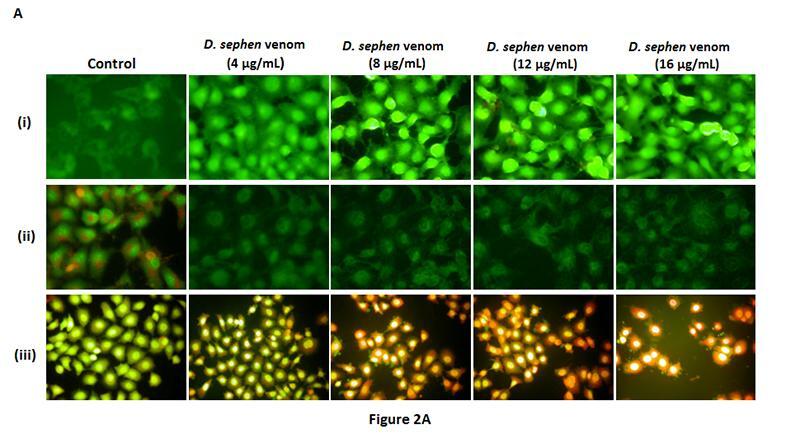


The correct version of the figure is:


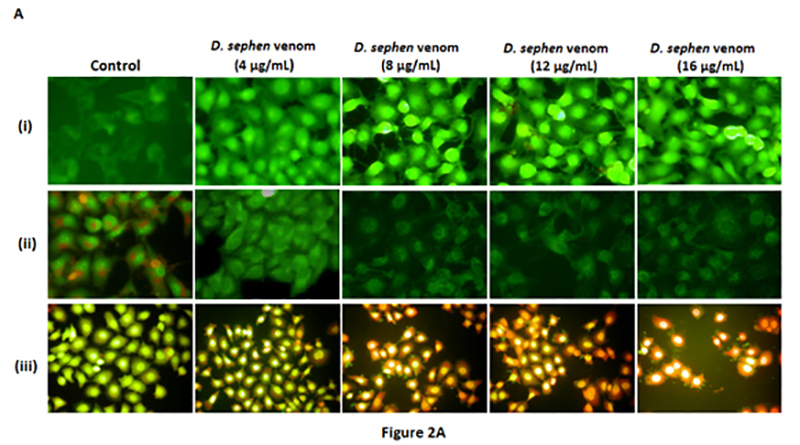


The correction does not affect the discussion or conclusions of the original article.

