# Supplementary material for: Antiproliferative activity of marine stingray Dasyatis sephen venom on human cervical carcinoma cell line
Source: J Venom Anim Toxins Incl Trop Dis. 2015 Oct 12;21:41. doi: 10.1186/s40409-015-0036-5 (PMC4603964; doi:10.1186/s40409-015-0036-5)
Supplement: Supplementary file 1 — Scientific classification and image ofD. sephen. (DOCX 105 kb) [file 40409_2015_36_MOESM1_ESM.docx]

**Identification**

Stingrays are dorsoventrally flattened fish with protruding eyes in the dorsal side and having one to four venomous stingers on the dorsum of an elongated, whip-like caudal appendage which are bilaterally serrated, dentinal caudal spine. The large, flag-like ventral fold on the tail is unique and key characteristic of *D. sephen* species (see figure below).

**Systematic position**

- Domain: [Eukaryota](http://zipcodezoo.com/Key/Animalia/Eukaryota_Domain.asp) – Whittaker & Margulis,1978
  - Phylum: [Chordata](http://zipcodezoo.com/Key/Animalia/Chordata_Phylum.asp) – Bateson, 1885 – Chordates
    - Class: [Chondrichthyes](http://zipcodezoo.com/Key/Animalia/Chondrichthyes_Class.asp)
      - Subclass: [Elasmobranchii](http://zipcodezoo.com/Key/Animalia/Elasmobranchii_Subclass.asp) - Shark-like fishes
        - Order: [*Rajiformes*](http://zipcodezoo.com/Key/Animalia/Rajiformes_Order.asp)

Family: [Dasyatidae](http://zipcodezoo.com/Key/Animalia/Dasyatidae_Family.asp) – Stingrays

Genus: [*Dasyatis*](http://zipcodezoo.com/Key/Animalia/Dasyatis_Genus.asp)

Specific name: *sephen*

Scientific name: *Dasyatis sephen* (Forsskal,1775)

**Common Names**

English: Cowtail stingray

French: Pastenague plumetée

**Synonyms**

*Dasyatis sephen* (Forsskål, 1775)

*Himantura fluviatilis* (Hamilton-Buchanan, 1822)

*Hypolophus sephen* (Forsskål, 1775)

*Raja sephen* Forsskål, 1775


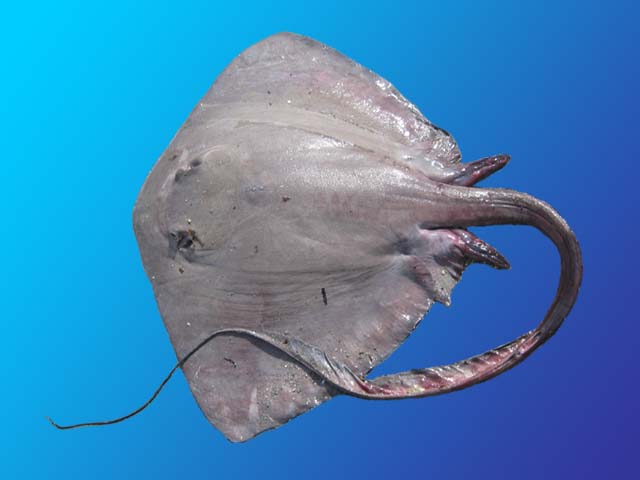

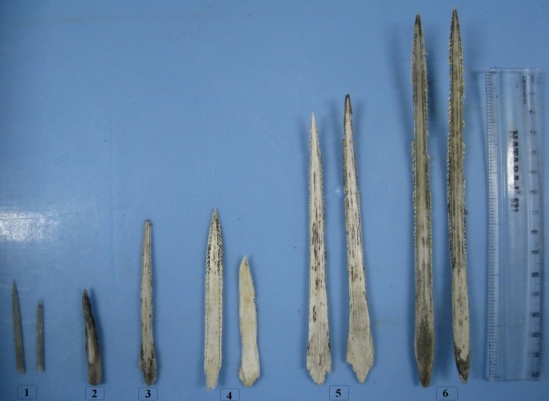


A

B

**Figure.**  (A) *D. sephen*. (B) *D. sephen* stinger.
